# Necrotizing Funisitis as an Intrauterine manifestation of Cryopyrin-Associated Periodic Syndrome: a case report and review of the literature

**DOI:** 10.1186/s12969-021-00578-2

**Published:** 2021-05-31

**Authors:** Kyoko Yokoi, Sachiko Minamiguchi, Yoshitaka Honda, Mizuho Kobayashi, Satoru Kobayashi, Ryuta Nishikomori

**Affiliations:** 1 Department of Pediatrics, Komaki City Hospital, 1-20, Jobushi, Aichi 485-8520 Komaki, Japan; 2 Department of Pediatrics, Nagoya West Medical Center, Nagoya, Japan; 3 Department of Diagnostic Pathology, Kyoto University Graduate School of Medicine, Kyoto, Japan; 4 Department of Pediatrics, Kyoto University Graduate School of Medicine, Kyoto, Japan; 5 Department of Diagnostic Pathology, Nagoya West Medical Center, Nagoya, Japan; 6 Department of Pediatrics and Child Health, Kurume University School of Medicine, Kurume, Japan

**Keywords:** cryopyrin associated periodic syndrome, neonatal-onset multisystem inflammatory disease/chronic infantile neurologic cutaneous and articular syndrome, funisitis, preterm

## Abstract

**Background:**

Cryopyrin-associated periodic syndrome (CAPS) is a life-long, autoinflammatory disease associated with a gain-of-function mutation in the nucleotide-binding domain, leucine-rich repeat family, pyrin domain containing 3 (*NLRP3*) gene, which result in uncontrolled production of IL-1β and chronic inflammation. Chronic infantile neurologic cutaneous and articular (CINCA) syndrome/neonatal-Onset multisystem inflammatory disease (NOMID) is the most severe form of CAPS. Although the first symptoms may be presented at birth, there are few reports on the involvement of the placenta and umbilical cord in the disease. Therefore, we present herein a preterm case of CINCA/NOMID syndrome and confirms intrauterine-onset inflammation with conclusive evidence by using fetal and placental histopathological examination.

**Case presentation:**

The female patient was born at 33weeks of gestation by emergency caesarean section and weighted at 1,514 g. The most common manifestations of CINCA/NOMID syndrome including recurrent fever, urticarial rash, and ventriculomegaly due to aseptic meningitis were presented. She also exhibited atypical symptoms such as severe hepatosplenomegaly with cholestasis. The genetic analysis of *NLRP3* revealed a heterozygous c.1698 C > G (p.Phe566Leu) mutation, and she was diagnosed with CINCA/NOMID syndrome. Further, a histopathological examination revealed necrotizing funisitis, mainly inflammation of the umbilical artery, along with focal neutrophilic and lymphocytic villitis.

**Conclusions:**

The necrotizing funisitis, which only involved the artery, was an unusual observation for chorioamnionitis. These evidences suggest that foetal inflammation, probably due to overproduction of IL-1β, caused tissue damage in utero, and the first symptom of a newborn with CINCA/NOMID.

## Background

Cryopyrin-associated periodic syndrome (CAPS) is a life-long, autoinflammatory disease associated with a gain-of-function mutation in the nucleotide-binding domain, leucine-rich repeat family, pyrin domain containing 3 (*NLRP3*) gene, which codes for cryopyrin [1]. Cryopyrin is a vital part of the inflammasome complex, which activates caspase-1 and causes subsequent production of interleukin-1 beta (IL-1β). Abnormalities in cryopyrin cause uncontrolled production of IL-1β, resulting in chronic inflammation and tissue damage in CAPS [2, 3]. CAPS comprise of three diseases with specific clinical features and disease activity; the mildest form of which is familial cold auto-inflammatory syndrome, Muckle-Wells syndrome is the moderate form, and the most severe form is chronic infantile neurologic cutaneous and articular (CINCA)/neonatal-onset multisystem inflammatory disease (NOMID) syndrome. All three forms are caused by the same gene, *NLRP3* [4, 5]. The prevalence of CINCA/NOMID syndrome is estimated to be 1 in 1,000,000, and approximately 40 people were diagnosed with CINCA/NOMID syndrome in Japan [6]. Although the first symptoms of CINCA/NOMID syndrome may be present at birth, there are few reports on the involvement of the placenta and umbilical cord in the disease [7]. Therefore, we present herein a preterm case of CINCA/NOMID that was complicated with the necrotizing funisitis which only involved the artery, which was confirmed by using fetal and placental histopathological examination.

## Case presentation

**Fig. 1:**
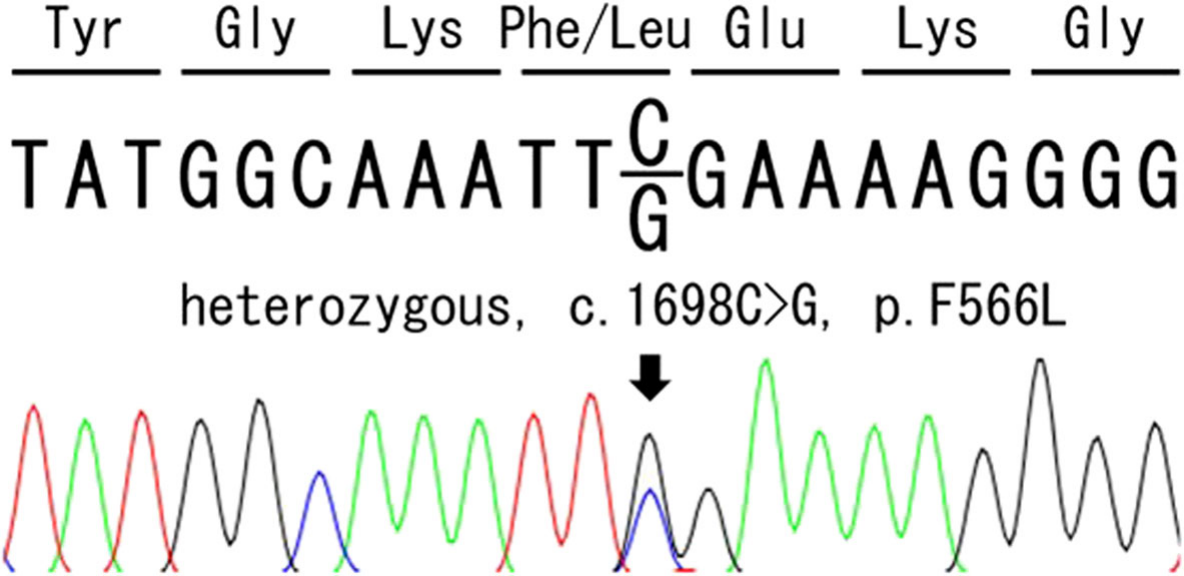
Chromatogram of the *NLRP3* gene mutation in the patient. The coding region of *NLRP3* genes were PCR amplified and sequences were determined by the conventional Sanger Sequencing as described previously [9]. The arrow indicates a missense mutation in exon 3 (c. 1698 C > G)

The female patient was born at 33weeks and 5 days of gestation by emergency cesarean section following presentation of pregnancy complication including polyhydramnios, spontaneous preterm labor, and elevated maternal C-reactive protein (CRP). Her parents were nonconsanguineous, and family history was unremarkable. Apgar scores were 5 at 1 min, and 7 at 5 min, with birthweight of 1,514 g (-1.8 SD), length of 42 cm (-0.74 SD), and head circumference of 28.3 cm (-1.29 SD). She presented with severe hepatosplenomegaly and mild ventriculomegaly at birth, as well as urticarial rash on the body and limbs after a few hours of birth. The blood examination at birth revealed CRP at 6.4 mg/dL, white blood cell count (WBC) of 34,650 /µL, red blood cell count of 323 × 10^4^ /µL, hemoglobin 12.7 g/dL and platelet 7 × 10^4^ /µL. In addition, the examination of the cerebrospinal fluid (CSF) showed a WBC cell count of 667 /µL (polymorphonuclear: mononuclear 4:1), glucose at 21 mg/dL, and protein at 191 mg/dL. No pathogenic bacteria were isolated from the patient, her CRP continued to increase to 15 mg/dL despite intensive antibiotics treatment. The neonatal-onset rash and aseptic meningitis, combined with persistent elevation of CRP suggested the diagnosis of auto-inflammatory disease, more specifically CAPS. Urinary excretion of mevalonic acid was not elevated, and the possibility of hyper IgD syndrome was excluded. After obtaining an informed consent from her parents, we performed genetic analysis of NLRP3 on her whole blood, which revealed heterozygous NLRP3 c.1698 C > G (p.Phe566Leu) variant that has been reported as disease-causing in infevers database (https://infevers.umai-montpellier.fr/web/search.php?n=4) (Fig. 1) [8]. Genetic analysis of NLRP3 was not performed for her parents since they refused to take the tests. Based on the clinical manifestations and genetic analysis, she was finally diagnosed with CINCA/NOMID.

Meanwhile, the hepatosplenomegaly with cholestasis gradually deteriorated to a total bilirubin of 5.4 mg/dL and direct bilirubin of 3.5 mg/dL, aspartate aminotransferase of 147 IU/L and alanine aminotransferase of 83 IU/L. Contrast-enhanced computed tomography of the abdomen was normal except severe hepatosplenomegaly. Specific IgM antibody serology tests against toxoplasmosis, cytomegalovirus, rubella virus, syphilis, and herpes simplex virus were negative. The plasma analysis of amino acids by high performance liquid chromatography was not characteristic of neonatal intrahepatic cholestasis caused by citrin deficiency such as elevated citrulline, tyrosine, phenylalanine, methionine and threonine. Bone marrow examination was performed, and no malignancy nor hemophagocytosis were observed. Oral ursodeoxycholic acid, fat-soluble vitamins and medium chain fatty acids containing milk (MCT milk) were started at 16 days of age, however, these treatments had no remarkable effect on her cholestasis.

At 70 days of age, after diagnosis was confirmed genetically, treatment with recombinant anti- human IL-1β monoclonal antibody (canakinumab) was started at a dose of 2.5 mg/kg. The rash immediately disappeared, and inflammatory markers slightly decreased after the first administration. Cholestasis slowly improved and ursodeoxycholic acid, fat-soluble vitamins and MCT milk were discontinued at the age of 4 months. We finally increased the dose of canakinumab stepwise up to 7.8 mg/kg every 4–6 weeks, and no further complications from the use of canakinumab were noted since the age of 7 months.

### The pathological findings of the placenta and umbilical cord

**Fig. 2:**
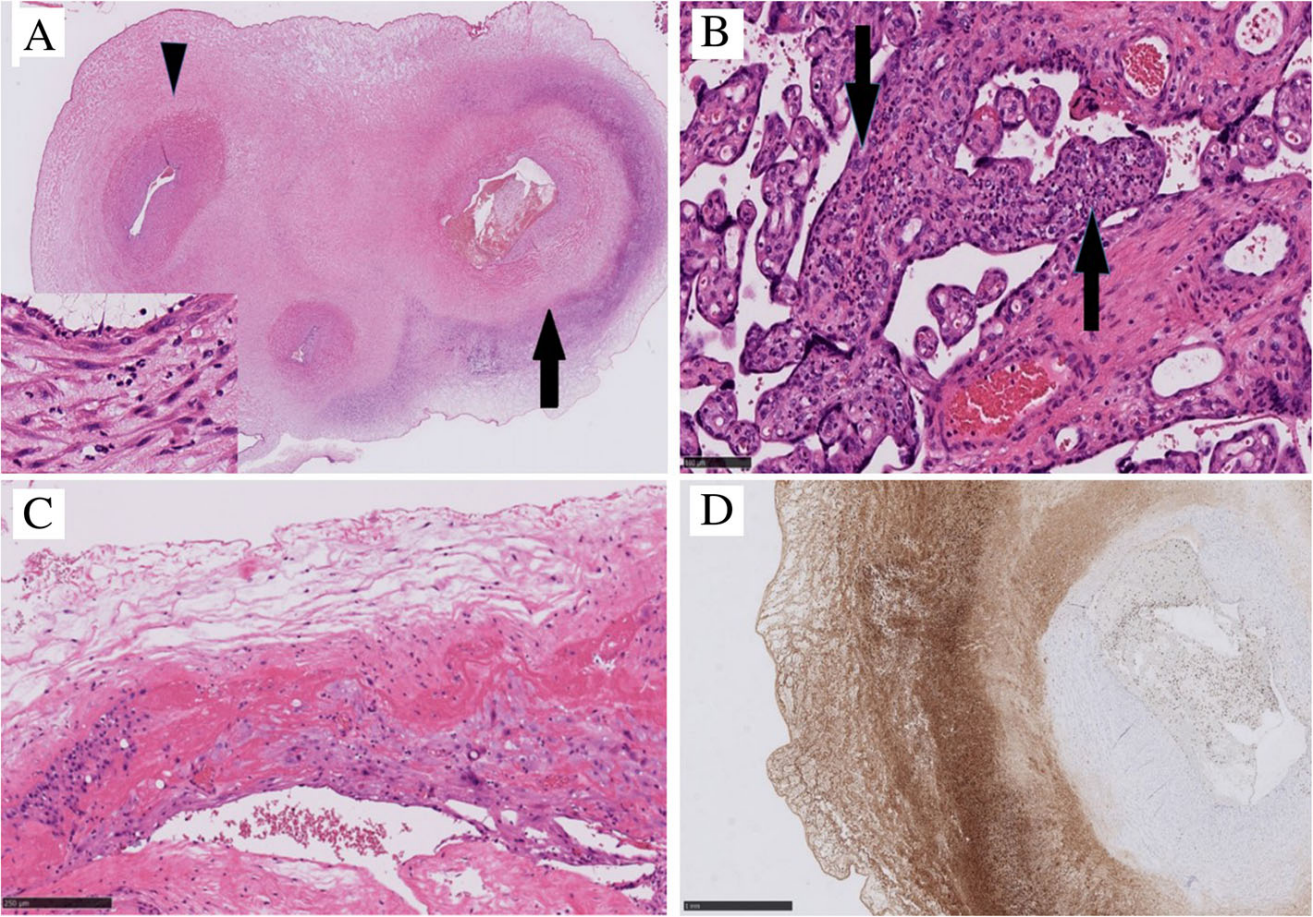
Microscopic findings of the placenta and umbilical cord. **A**. Necrotizing funisitis: the umbilical cord showed ring-shaped infiltration of inflammatory cells around the artery (arrow and inset), but not in the vein (arrow head). (Hematoxylin an eosin (HE) staining). **B**. Focal neutrophilic and lymphocytic villitis: destructive inflammation of chorionic villus (arrow), followed by mild collapse of villous vessels (HE staining). **C**. The chorioamnion without neutrophilic infiltration. **D**. Immunohistochemical staining of myeloperoxidase: the inflammation around the umbilical artery and perivascular Wharton’s jelly caused mainly by macrophages and neutrophils

The umbilical cord was found to be edematous and contained yellow discoloration on the surface. Moreover, the placenta was friable, and weighted 615 g, which is a very heavy placenta, greater than 90th percentile for the gestational age. The histopathological examination revealed necrotizing funisitis, namely inflammation of the umbilical cord (Fig. 2 A), and along with focal neutrophilic and lymphocytic villitis (Fig. 2B). The neutrophils were not infiltrated in the chorioamnion (Fig. 2 C). While inflammatory cells presented ring-shaped infiltration on the surface of one of the umbilical arteries and within the Wharton’s jelly, there was no inflammatory response in umbilical vein (Fig. 2 A). These inflammatory cells were mainly neutrophils and macrophages, demonstrated by immunohistochemical positive for myeloperoxidase (Fig. 2D), a portion of which shows CD68 positivity.

## Discussion

CINCA/NOMID syndrome is the most severe form of CAPS, which is characterized by neonatal onset and presents with recurrent fever and urticarial rash. Without appropriate treatments, chronic inflammation continues, causing neurological manifestations, arthritis, arthropathy, and uveitis [10, 11]. In Japan, only canakinumab has been approved since 2011 for the treatment of all three forms of CAPS [12]. The definite diagnosis for CINCA/NOMID relies mainly on genetic analysis of NLRP3, which has more than 100 reported pathogenic or likely pathothenic variants [8]. Further, approximately 28 ~ 35 % of all the CINCA/NOMID patients carry an *NLRP3* mutation in somatic mosaicism state [9, 13], which causes patients to present with milder neurologic symptoms compared to those with germline mutation of the same variant. However, no additional differences in symptoms was observed in age at disease onset, skin symptoms and joint involvement, whereas, the p.Phe566Leu variant was previously reported as a CAPS disease-causing mutation within somatic mosaicism cohort, which present with meningitis, arthritis and walking disability [9]. Our patient was found to have p.Phe566Leu variant as a heterozygous germline mutation, which might explain the most severe phenotype including continuous inflammatory biological markers and atypical symptoms.

Furthermore, hepatosplenomegaly with normal liver functions was occasionally described in CINCA/NOMID patients, and liver biopsy demonstrated non-specific presentation for chronic inflammation in the parenchyma and subcapsular area in such a case [7]. However, the patients with cholestasis were only reported by Paccaud et al. [14]. Their patient was identified as heterozygous for the p.Glu567Lys mutation and had many similar characteristics to our case. First, both cases were born at preterm 33 weeks of gestation, complicated with polyhydramnios. Second, hepatosplenomegaly with cholestasis was initially severe and gradually improved in parallel to decreasing inflammatory reaction. Following detailed examination, our patient did not have any other disease associated with cholestasis, and her symptoms gradually improved as CRP decreased. The observed chronic inflammation caused the liver damage in preterm neonate, resulting in hepatosplenomegaly and cholestasis. It is of great interest whether the treatment with canakinumab is able to subside the occurrence of complications on hepatobiliary system.

The preterm, intrauterine growth retardation, polyhydramnios, and placental and umbilical abnormalities were often presented in CINCA/NOMID syndrome. It has been reported that histological examination of the placenta from CINCA/NOMID syndrome has revealed vascular thrombosis, microcalcification and polymorphonuclear cell infiltrates [7, 14]. Table 1 describes the intrauterine manifestation of CINCA/NOMID syndrome patients in previous reports. Though polyhydramnios has been reported [7, 14], making our case the fourth, it was not possible to perform the detailed search for polyhydramnios due to an emergency cesarean section.

**Table 1:** Intrauterine manifestation of CINCA/NOMID patients in previous reports

Intrauterine manifestation	Reference	The number of cases	Mutation	Commentary
Abnormal amount of amniotic fluid	Prieur AM, et al. (1987) [7]	2	N.D.	Polyhydramnios
	Caroli F, et al. (2006) [15]	1	M406I	Oligohydramnios
	Paccaud Y, et al. (2014) [14]	1	E567K	Polyhydramnios
	Our case	1	F566L	Polyhydramnios
Histological findings of placenta and umbilical cord	Prieur AM, et al. (1987) [7]	1	N.D.	Placenta with thrombosis and calcification Small villi and umbilical cord with polymorphonuclear infiltration
	Paccaud Y, et al. (2014) [14]	1	E567K	Umbilical cord infection
	Madison E, et al. (2019) [16]	1	N.D.	Necrotizing funisitis
	Our case	1	F566L	Neutrophilic and lymphocytic villitis Necrotizing umbilical arteritis

N.D.: not described

Necrotizing funisitis, which is often associated with increased rates of stillbirth, perinatal infection, and preterm delivery, is sometimes accompanied with congenital syphilis [17]. In our case, the possibility of congenital syphilis was excluded because of the absence of the specific IgM antibodies. Acute chorioamnionitis provides evidence of a maternal host response, whereas funisitis represents a foetal inflammatory response [18]. These inflammatory responses are characterized by the infiltration of neutrophils and release of cytokines [18, 19]. Moreover, funisitis is often associated with inflammation of the chorioamnion due to ascending infection. The inflammation of the umbilical vessels typically begins in the vein (phlebitis) and is followed by involvement of the arteries (arteritis) and the Wharton’s jelly [20]. Since our case was not complicated with severe chorioamnionitis, it is likely that the necrotizing funisitis that only involved the artery, was not secondary to chorioamnionitis, but due to foetal origin inflammation. These evidences suggest that foetal inflammation, probably due to overproduction of IL-1β by CINCA/NOMID, might cause necrotizing funisitis in utero. According to previous reports, all histopathological findings revealed funisitis or inflammation of umbilical cords, which might be quite usual manifestation in CINCA/NOMID syndrome (Table 1).

Moreover, our patient was complicated with villitis, namely inflammation of chorioamniotic villi. Some villitis is caused by an infectious agent, however, most chronic inflammatory lesions are of unknown aetiology [16] [21]. Excessive foetal IL-1β might have injured the villi to induce a maternal inflammatory response, causing the elevated maternal CRP before the emergency caesarean section.

## Conclusions

We emphasize that the histopathological examination of placenta and umbilical cord can be one of the clues indicating intrauterine-onset inflammation and the first symptom of a newborn with CINCA/NOMID.

## Data Availability

The data are available from the corresponding author on reasonable request.

## Abbreviations

CAPS: cryopyrin-associated periodic syndrome
NLRP3: nucleotide-binding domain, leucine-rich repeat family, pyrin domain containing 3
IL-1β: interleukin-1 beta
CINCA/ NOMID: chronic infantile neurologic cutaneous and articular syndrome/neonatal-onset multisystem inflammatory disease
CRP: c-reactive protein
WBC: white blood cell
MCT milk: medium chain fatty acids containing milk
